# *Keratinocyte-associated protein 3* plays a role in body weight and adiposity with differential effects in males and females

**DOI:** 10.3389/fgene.2022.942574

**Published:** 2022-09-21

**Authors:** Alexandria M. Szalanczy, Emily Goff, Osborne Seshie, Aaron Deal, Michael Grzybowski, Jason Klotz, Chia-Chi Chuang Key, Aron M. Geurts, Leah C. Solberg Woods

**Affiliations:** ^1^ Department of Internal Medicine, School of Medicine, Wake Forest University, Winston Salem, NC, United States; ^2^ Department of Physiology, Medical College of Wisconsin, Milwaukee, WI, United States

**Keywords:** obesity, genetics, rat model, fat mass, sex differences

## Abstract

Despite the obesity crisis in the United States, the underlying genetics are poorly understood. Our lab previously identified *Keratinocyte-associated protein 3, Krtcap3,* as a candidate gene for adiposity through a genome-wide association study in outbred rats, where increased liver expression of *Krtcap3* correlated with decreased fat mass. Here we seek to confirm that *Krtcap3* expression affects adiposity traits. To do so, we developed an *in vivo* whole-body *Krtcap3* knock-out (KO) rat model. Wild-type (WT) and KO rats were placed onto a high-fat (HFD) or low-fat diet (LFD) at 6 weeks of age and were maintained on diet for 13 weeks, followed by assessments of metabolic health. We hypothesized that *Krtcap3*-KO rats will have increased adiposity and a worsened metabolic phenotype relative to WT. We found that KO male and female rats have significantly increased body weight versus WT, with the largest effect in females on a HFD. KO females also ate more and had greater adiposity, but were more insulin sensitive than WT regardless of diet condition. Although KO males weighed more than WT under both diet conditions, there were no differences in eating behavior or fat mass. Interestingly, KO males on a HFD were more insulin resistant than WT. This study confirms that *Krtcap3* plays a role in body weight regulation and demonstrates genotype- and sex-specific effects on food intake, adiposity, and insulin sensitivity. Future studies will seek to better understand these sex differences, the role of diet, and establish a mechanism for *Krtcap3* in obesity.

## Introduction

Global obesity rates have nearly tripled in the last 50 years, and in the United States adult obesity rates are expected to reach 50% by 2030 (Ward et al., 2019; Hales et al., 2020). Obesity is influenced by both environment and genetics (Abadi et al., 2017; Yengo et al., 2018; Pulit et al., 2019), but the genetic architecture underlying the disease is poorly understood. Current estimates place narrow-sense heritability of body mass index (BMI), a marker of obesity, at approximately 40%–50% (Bouchard, 2021). Although many genetic loci have been identified in human genome wide association studies (GWAS), many of the causal genes are unknown and require functional validation studies. Additionally, known loci explain only a small portion of heritability, indicating more genes have yet to be found (Yengo et al., 2018; Pulit et al., 2019; Rohde et al., 2019). In most GWAS analyses, multiple genes fall under a single locus, so it is important to conduct validation studies to confirm the underlying causal genes. Further, because GWAS do not make *a priori* assumptions about the biology between the genetics and disease, studies must be performed to identify how these genes contribute to obesity. Importantly, as more variants are identified, there will be diminishing effect sizes, as most common obesity variants will impact weight very subtly—even as small as only a few grams in humans (Bouchard, 2021). That being said, better understanding the genes that impact obesity may lead to improved treatment options for patients suffering with obesity.

Our laboratory has used the outbred heterogeneous stock (HS) rat as a complementary method for genetic mapping of obesity traits (Keele et al., 2018; Chitre et al., 2020) and have demonstrated functional concordance in adiposity-associated adipose tissue transcripts between HS rats and humans (Crouse et al., 2022). HS rats are outbred from eight inbred founder strains (Hansen and Spuhler, 1984), allowing genetic fine-mapping to Mb intervals (Solberg Woods and Palmer, 2019). Previously, we used mediation analysis to identify *Keratinocyte-associated protein 3* (*Krtcap3*) as a candidate gene for visceral fat mass within a quantitative trait locus (QTL) on rat chromosome 6 (Keele et al., 2018). In the rat model, liver *Krtcap3* expression was negatively correlated with fat mass, suggesting a protective role against obesity (Keele et al., 2018). Another group also found that *Krtcap3* is a potential pleiotropic gene for obesity, type 2 diabetes (T2D), and dyslipidemia in humans (Chen et al., 2018), indicating translational relevance. Beyond these two investigations, *Krtcap3* has not been well-studied, and there is no published research at the time of this article that describes a function for the protein KRTCAP3. This study is the first to investigate *Krtcap3* as an adiposity gene, and serves to validate the previous GWAS findings.

To determine the role of *Krtcap3* on adiposity, we knocked-out *Krtcap3* expression in an *in vivo* Wistar-Kyoto (WKY) rat model. The WKY haplotype at the chromosome 6 QTL in HS rats conferred susceptibility to high liver *Krtcap3* expression and decreased fat mass (Keele et al., 2018). WKY rats also have naturally high levels of liver *Krtcap3* expression compared to the other HS founder strains. Wild-type (WT) and *Krtcap3* knock-out (KO) rats were placed on either a low-fat (LFD) or high-fat diet (HFD) and multiple metabolic traits were measured. We show that *Krtcap3-*KO rats have increased body weight in both sexes, but increased food intake and fat mass only in female KO rats. There were also differential effects by sex on insulin sensitivity, where KO females were more insulin sensitive than WT females, but KO males on a HFD were more insulin resistant than WT controls. This work validates the previous GWAS results by confirming the role of *Krtcap3* in adiposity, and demonstrates sex-specific differences on its impact on food intake, adiposity, and insulin sensitivity. Future work will uncover a mechanistic role for *Krtcap3* and seek to understand the sex differences.

## Materials and methods

### Animals

**FIGURE 1:**
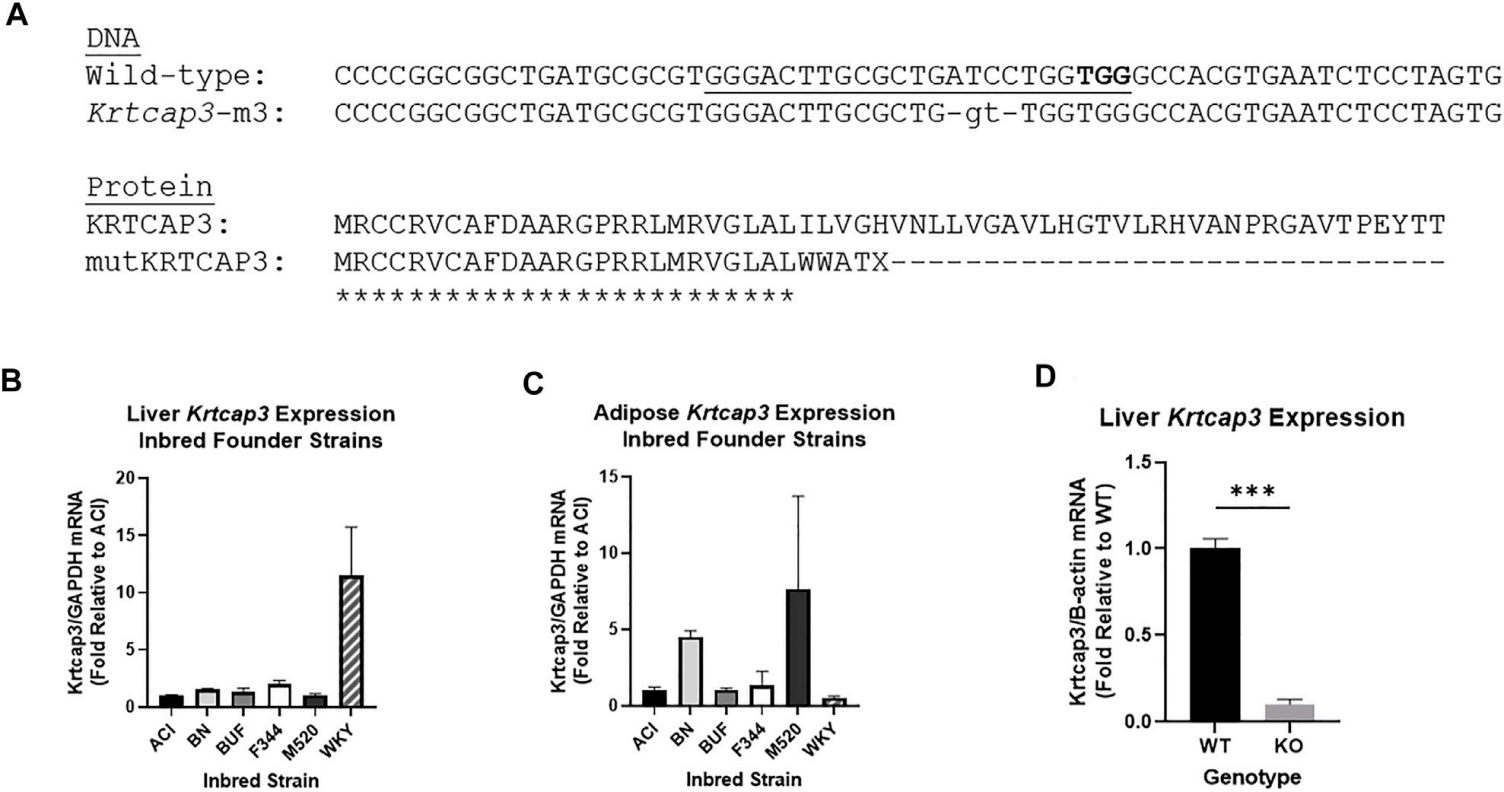
Development and confirmation of *Krtcap3* knock-out (KO) rats and tissue expression analyses. **(A)** CRISPR-Cas9 mutagenesis was used to target the second exon (underlined sequence, protospacer adjacent motif in bold), resulting in a 4 base-pair deletion and 2-based pair insertion (lowercase). This results in a predicted frameshift after 25 amino acids and premature termination of the normal protein sequence (only the first 60 of 240 amino acids shown). Asterisks indicate identical amino acids. **(B)** RNA was extracted from liver tissue of available HS inbred founder strains to compare *Krtcap3* expression. Available strains are ACI/Eur, BN/SsHsd, F344/NHsd, M520/NMcwi, WKY/NHsd. Fold change was calculated relative to ACI *Krtcap3* expression. **(C)** RNA was extracted from retroperitoneal adipose tissue of the same inbred founder strains to compare *Krtcap3* expression. Fold change was calculated relative to ACI *Krtcap3* expression. **(D)** RNA was extracted from liver tissue of adolescent WKY wild-type (WT) and KO rats (*n* = 3 per genotype, mixed sex) and *Krtcap3* expression analyzed to assess the success of the *Krtcap3*-KO. Fold change was calculated relative to WT *Krtcap3* expression. ***p < 0.001.

To develop the *Krtcap3*-KO (WKY-Krtcap3^em3Mcwi^) we injected a CRISPR targeting the sequence GGG​ACT​TGC​GCT​GAT​CCT​GG into Wistar-Kyoto (WKY/NCrl; RGD_1358112) rat embryos (Figure 1A). The WKY rat was chosen as the background strain for the reasons outlined above, and we demonstrate here that the WKY strain has high *Krtcap3* expression in liver tissue relative to other inbred strains, but lower expression in adipose tissue (Figures 1B,C). The resulting mutation was a net 2 base pair deletion, which led to a frameshift mutation and early termination of the KRTCAP3 protein. Founder animals at the Medical College of Wisconsin (MCW) were genotyped by the Cel-1 assay (Kulinski et al., 2000) and confirmed by Sanger sequencing. Founders were then backcrossed to the parental WKY strain and subsequent litters were genotyped by fluorescent genotyping.

Three heterozygous (HET) breeding pairs were used to establish a breeding colony at Wake Forest University School of Medicine (WFSoM). Rats were housed in standard caging at 22°C in a 12 h light and 12 h dark cycle (dark from 18:00 to 6:00) at standard temperature and humidity conditions and given *ad libitum* access to water. Breeders were given standard chow diet (Lab Diet, Prolab RMH 3000, Catalog #5P00) while experimental rats were placed on experimental diet, as described below. We used real-time quantitative PCR (rt-qPCR), as described below, to verify significantly decreased *Krtcap3* expression levels in the liver of 5-week old *Krtcap3*-KO rats relative to WT (T_4_ = 14.44, *p* = 6.67e-5) (Figure 1D). We were unable to validate these findings using a Western blot due to the lack of a reliable KRTCAP3 antibody.

### Genotyping

Once the colony was established at WFSoM, experimental WT and KO rats were genotyped one of two ways. The first was fluorescent based fragment analysis at MCW using the ABI 3730 capillary sequencer followed by analysis in Genemapper software. The second method, at WFSoM, was to amplify *Krtcap3* over the mutation site, and then digest the samples with the restriction enzyme MboI. WT samples would be digested into smaller strands while KO samples, which lacked the MboI binding site due to the mutation, would remain the same size as the original PCR product. When run out on a 1.5% DNA agarose gel, WT and KO samples were distinguished by their banding pattern. The same primer for *Krtcap3* amplification was used for genotyping at MCW and at WFSoM (Supplementary Table S1).

### Real-time quantitative PCR

We compared *Krtcap3* expression in liver and adipose tissue between available inbred founder strains (ACI/Eur, BN/SsHsd, F344/NHsd, M520/NMcwi, WKY/NHsd) (Figures 1B,C). RNA was extracted by Trizol and expression was measured using rt-qPCR. *GAPDH* (Supplementary Table S1) was used as the housekeeping gene and fold change of *Krtcap3* (Supplementary Table S1) transcript was calculated by the following equation:

$$Transcript=2^{-∆∆Ct}$$

Where ΔCt was the difference between the crossing threshold of *Krtcap3* and *GAPDH*, and ΔΔCt the difference between each sample ΔCt and the average ΔCt of the ACI founder strain.

To verify that the KO reduced *Krtcap3* expression, we collected liver tissue from adolescent WKY WT and KO rats (*n* = 3 per genotype, mixed sex). RNA was extracted by Trizol and expression was measured using rt-qPCR. *β-actin* (Supplementary Table S1) was used as the housekeeping gene, and fold change of *Krtcap3* transcript was calculated by the equation given above, but where ΔCt was the difference between the crossing threshold (Ct) of *Krtcap3* and *β-actin*, and ΔΔCt the difference between each sample ΔCt and the average WT ΔCt.

To compare differences in liver *Krtcap3* expression between adult males and females, we extracted RNA by Trizol from liver tissue collected at euthanasia from WT animals in the present study (see below). *β-actin* was used as the housekeeping gene, and ΔΔCt the difference between each sample ΔCt and the average ΔCt of WT LFD male rats.

### Study design

**FIGURE 2:**
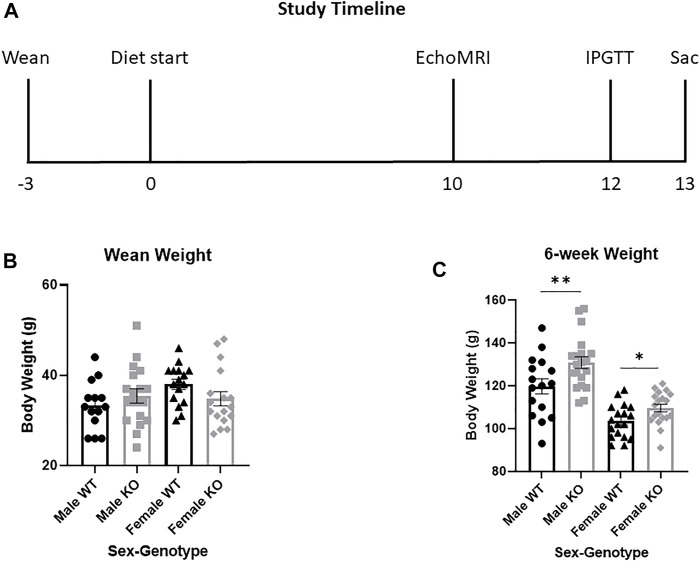
Study design and body weight in wild-type (WT, black) and *Krtcap3* knock-out (KO, gray) rats prior to diet start. **(A)** Timeline outlining study design, with weeks relative to diet start shown. Metabolic phenotyping included EchoMRI analysis (EchoMRI), an intraperitoneal glucose tolerance test (IPGTT), and euthanasia (Sac). **(B)** At time of wean at 3 weeks of age, there were no differences in body weight between WT males (circle) and KO males (square), nor between WT females (triangle) and KO females (diamond). By diet start at 6 weeks of age, however, **(C)** KO rats of both sexes were significantly heavier than WT controls. *p < 0.05, **p < 0.01.

Experimental rats were weaned at 3 weeks of age and placed two per cage in same-sex, same-genotype cages. Prior to diet start, experimental rats were maintained on the same diet as breeders (Lab Diet, Prolab RMH 3000, Catalog #5P00). At 6 weeks of age, rats were weighed and began either a HFD (60%kcal fat; ResearchDiet D12492) or a sucrose-matched LFD (10%kcal fat; ResearchDiet D12450J) (Supplementary Table S2). Hereafter rats will be referred to by genotype-diet-sex, such that WT males consuming HFD are called WT HFD males. Rats were allowed access to diet *ad libitum* with body weight and food intake recorded weekly starting at 6 weeks of age. Rats were on diet for 13 weeks, with metabolic phenotyping tests beginning after 10 weeks on diet and euthanasia after 13 weeks on diet, as described (Figure 2A). We saved 8–11 rats per genotype-diet-sex group. Due to COVID-19 shut-downs, we were unable to complete the metabolic phenotyping procedure in all rats (Supplementary Table S3). All experiments were performed using a protocol approved by the Institutional Animal Care and Use Committee at WFSoM.

### Metabolic phenotyping

After 10 weeks on diet, rats went through EchoMRI (EchoMRI LLC, Houston, TX) analysis, which precisely measures fat mass and lean mass of live rats. Non-fasted rats were weighed before analysis then scanned for 2 min in triplicate.

After 12 weeks on diet, rats were fasted 16 h overnight before being administered an intraperitoneal glucose tolerance test (IPGTT). We collected blood glucose (Contour Next EZ) and serum for subsequent insulin analysis from a tail nick at fasting and 15, 30, 60, and 90 min after a 1 mg/kg glucose injection. To calculate a rat’s response to the glucose challenge, the glucose area-under-the-curve (AUC), we used the following equation:

$$Glucose\;AUC=\sum \frac{\left({SG}_{t}+{SG}_{t+1}\right)\times ∆\min\;}{2}$$

Where serum glucose (SG) was measured at t = 0, 15, 30, 60, and 90 min.

### Tissue harvest

After 13 weeks on diet, rats were euthanized *via* live decapitation after a 16 h overnight fast. Weight gain was calculated as the difference between the final body weight of the rats following the fast and the body weight at study start (weight at 19 weeks of age—weight at 6 weeks of age). Trunk blood was collected and serum saved and stored for lipid and biochemistry analysis. Body length from nose to anus was measured with a ruler. The brain, fat pad tissues [retroperitoneal (RetroFat), gonadal, and omental/mesenteric fat (OmenFat)] and a lobe of liver were dissected, weighed, and snap-frozen. The kidneys and the heart were also weighed and snap-frozen. Pancreas was weighed, saved on ice in acid ethanol, chopped by scissors the same day, and processed 48 h later to be stored at −20 C for subsequent measurement of whole pancreas insulin content (WPIC). Additional sections of liver and RetroFat were saved in 10% neutral-buffered formalin for histology. Due to research facility restrictions at the onset of COVID-19, the tissue harvest protocol had to be shortened, and OmenFat and pancreas for WPIC were not collected from all animals (Supplementary Table S3). Liver tissue from WT rats was used to measure *Krtcap3* expression in adult male and female rats.

### Insulin

We used ultrasensitive ELISA kits (Alpco Ref # 80-INSRTU-E10) to analyze serum insulin from the IPGTT and WPIC. Insulin response to the IPGTT, insulin AUC, was calculated with the same equation as glucose AUC. WPIC was normalized to pancreas weight. Homeostatic model assessment for insulin resistance (HOMA-IR) was used to approximate insulin resistance and was calculated by the formula (Sarafidis et al., 2007):

$$HOMA\_IR=\frac{fasting\;glucose\times fasting\;insulin}{22.5}$$

### Serum and liver analyses

Undiluted serum from each rat was sent to IDEXX BioAnalytics for analysis on a custom chemistry panel (62761), including measurements of triglycerides, total cholesterol, alkaline phosphatase (ALP), aspartate transaminase (AST), alanine aminotransferase (ALT), and albumin. Lipids were also extracted from liver tissue to measure triglyceride (Wako Diagnostics L-Type TG M) (Folch et al., 1957) and cholesterol (Pointe Scientific Cholesterol Liquid Reagents).

### Histology

We chose representative WT and KO samples from the HFD female group, where we saw the largest phenotypic difference in RetroFat (*n* = 5 per genotype). The Wake Forest Comparative Pathology Lab stained RetroFat samples for hematoxylin and eosin. Slides were electronically scanned by Wake Forest Virtual Microscopy Core, then analyzed with Visiopharm software (Hoersholm, Denmark). The area of approximately 1,000 adipocytes per slide (three slides per rat) were measured within at least three regions of interest per slide and plotted in a frequency distribution assessing adipocyte size. Only adipocytes with areas between 300 μm^2^ and 15,000 μm^2^ were considered.

### Statistical analysis

Unless otherwise specified, all data were analyzed in R (1.4.1103) with males and females analyzed separately. Outliers were assessed by Grubbs’ test and if *p* < 0.05, the suspected outlier was removed. Data were transformed to reflect a normal distribution (Supplementary Table S4). Homogeneity of variance was assessed by the Bartlett test. Wean weight and 6-week body weight between WT and KO rats were assessed with a Student’s *t*-test. Growth curves and cage food intake were assessed with a two-way repeated measures ANOVA, where the effect of genotype and diet were examined over time. If we saw a significant three-way interaction, data were separated by diet to analyze the effect of genotype over time. To measure cumulative energy consumed, the calories consumed per week per cage were calculated, and each week summed with the previous time points. A simple linear regression was fitted for each group and the slopes were tested in PRISM to determine if they were significantly different. We initially compared slopes from all diet and genotype groups within sex. Upon seeing large differences in slope between diet groups, we separated by diet to determine the impact of genotype. With the exception of histology data, all other metabolic data were analyzed by a two-way ANOVA, where the factors were genotype and diet. If there was a significant interaction, the analysis was split by diet condition and a Student’s *t*-test assessed the effect of genotype. We assessed the frequency distributions of adipocyte size between WT and KO rats by sorting adipocyte areas into bins from 0 to 15,000 μm^2^ in increments of 500 μm^2^. The frequency of each bin was calculated and the frequency distributions plotted. Data were analyzed by a two-way ANOVA, with the factors genotype and bin size.

Expression differences between adolescent WT and KO rats were assessed with a Student’s *t*-test. Expression differences between adult male and female rats were assessed by a two-way ANOVA, where the factors were diet and sex.

## Results

### Male and female knock-out rats are heavier than wild-type at 6 weeks of age

Although there were no differences in wean weight between WT and KO males or females (Figure 2B), by 6 weeks of age KO males and females were heavier than WT controls (T_34_ = 2.51, *p* = 0.0085; T_35_ = 2.43, *p* = 0.010, respectively) (Figure 2C). At 6 weeks of age, when rats began experimental diet, there were no significant differences in body weight between animals selected for HFD or LFD, respective to their sex and genotype.

### Male knock-out rats are heavier than wild-type with no difference in food consumption

**FIGURE 3:**
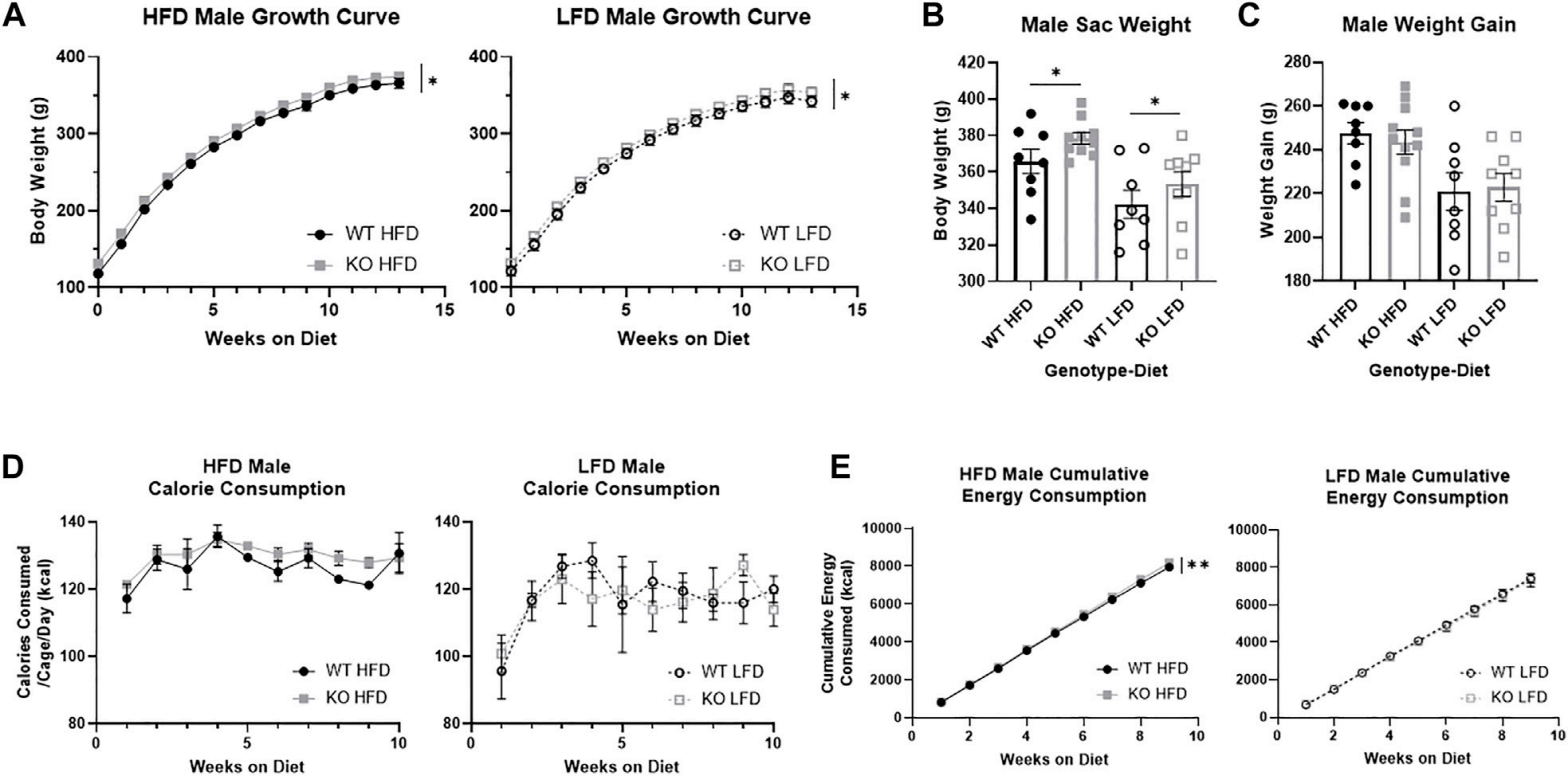
Body weight growth and energy consumption in male wild-type (WT; black circle) and *Krtcap3* knock-out (KO; gray square) rats. Rats were weighed weekly throughout the study, and final weight and overall weight gain were recorded. Calorie consumption was recorded for the first 10 weeks on diet, and cumulative energy consumption calculated for the first 9 weeks on diet. To highlight genotype-driven differences, figures are separated by diet, high-fat diet (HFD; filled) and low-fat diet (LFD; empty). **(A)** KO males were consistently heavier than WT males (circle) on both diets. Significance *p < 0.05 represents a main effect of genotype over time across both diets. **(B)** At euthanasia, KO males were heavier than WT males, where *p < 0.05 shows a main effect of genotype across both diets. **(C)** However, there were no differences in weight gain between WT and KO males. **(D)** There were no differences by genotype in caloric intake, yet. **(E)** KO HFD males had a greater rate of caloric intake than WT HFD males while there were no differences by genotype in LFD males. **p < 0.01 represents a significant difference in the slopes of the lines. Diet effects are reported in the “Results” section.

We measured differences in weight by genotype the full 13 weeks on diet, and found that KO males were heavier than WT males (F_1, 32_ = 4.44, *p* = 0.043; Figure 3A) and HFD males were heavier than LFD (F_1, 32_ = 4.68, *p* = 0.038). At study completion, KO male rats were heavier than WT (F_1, 32_ = 4.36, *p* = 0.045; Figure 3B), and HFD males were heavier than LFD (F_1, 32_ = 16.36, *p* = 3.1e-4), with no interactions between genotype and diet. Interestingly, there were no differences in weight gain between WT and KO males (F_1, 31_ = 0.001, *p* = 0.97; Figure 3C), indicating that the slight increase in body weight in KO at the start of the study was maintained throughout the study. As expected, HFD males gained more weight from diet start to study completion than LFD males (F_1, 31_ = 15.54, *p* = 4.3e-4).

During the first 10 weeks on diet, there were no differences in gross food intake by genotype (Supplementary Figure S1A), but LFD males ate significantly more than HFD males (F_1, 11_ = 120.18, *p* = 2.93e-7). Correspondingly, while there were no differences in energy consumption by genotype (Figure 3D), due to differences in energy content between the diets LFD males consumed fewer kcal than HFD males (F_1, 9_ = 10.53, *p* = 0.01). We measured cumulative energy consumption for the first 9 weeks of diet, and interestingly saw that KO HFD males had an greater rate of consumption compared to WT HFD males (F_1, 68_ = 8.69, *p* = 0.0044; Figure 3E), though there were no differences in LFD males.

### Female knock-out rats are heavier and eat more than wild-type

**FIGURE 4:**
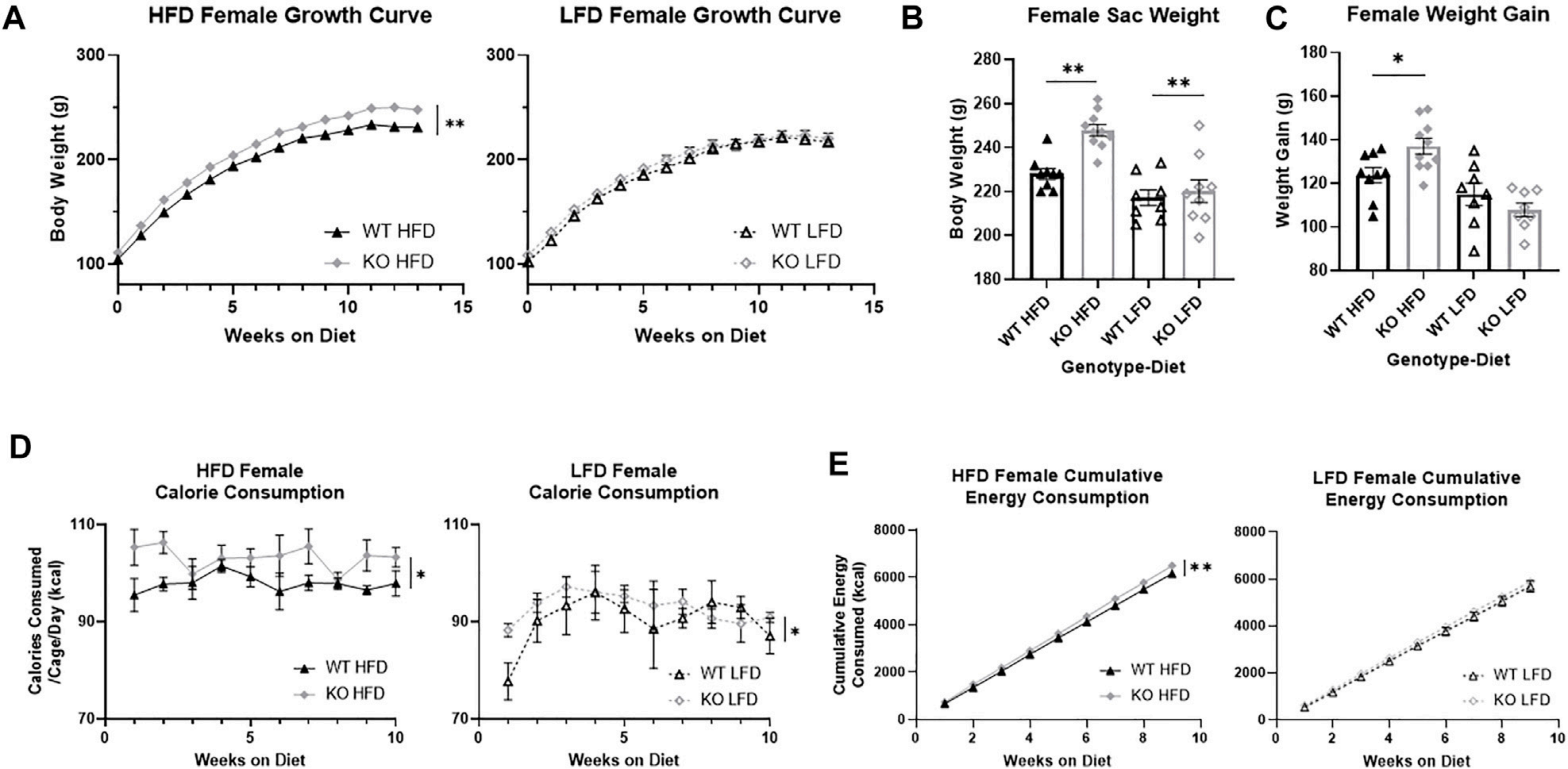
Body weight growth and energy consumption in female wild-type (WT; black triangle) and *Krtcap3* knock-out (KO; gray diamond) rats. Rats were weighed weekly throughout the study, and final weight and overall weight gain were recorded. Calorie consumption was recorded for the first 10 weeks on diet, and cumulative energy consumption calculated for the first 9 weeks on diet. To highlight genotype-driven differences, figures are separated by diet, high-fat diet (HFD; filled) and low-fat diet (LFD; empty). **(A)** In females, there was a three-way interaction between genotype, diet, and time. When data were analyzed separately by diet condition, KO females on a HFD weighed more than WT counterparts, with no difference in weight between WT and KO LFD females. **p < 0.01 for the effect of genotype over time in the HFD condition. **(B)** At euthanasia, KO females of both diets had a higher body weight than WT females, with no interaction between genotype and diet. **p < 0.01 represents main effect of genotype. **(C)** There was an interaction between genotype and diet for weight gain, where KO HFD females gained more weight than WT HFD females, with no differences in LFD females. *p < 0.05 represents the effect of genotype in the HFD condition. **(D)** KO females consumed more calories over time than WT females, where *p < 0.05 represents a main effect of genotype over time across both diets. **(E)** KO HFD females had a greater rate of caloric intake than WT HFD females, but there were no differences by genotype in LFD females. **p < 0.01 represents a significant difference in the slopes of the lines. Diet effects are reported in the “Results” section.

Over the course of the study, KO females were significantly heavier than WT females (F_1, 32_ = 11.54, *p* = 0.0018; Figure 4A) and HFD females were heavier than LFD (F_1, 32_ = 16.22, *p* = 3.2e-4). Visually there is a larger effect in KO HFD females relative to all other groups, which is supported by a significant three-way interaction between genotype, diet, and time (F_13, 416_ = 2.69, *p* = 0.0012). When separated by diet, KO HFD females weighed significantly more than WT (F_1, 17_ = 18.44, *p* = 4.9e-4), with no differences by genotype in LFD females. At the end of the study, KO female rats were significantly heavier than WT (F_1, 33_ = 9.67, *p* = 0.0039; Figure 4B) and HFD females were heavier than LFD (F_1, 33_ = 26.58, *p* = 1.16e-5), with no interactions between genotype and diet. When we measured weight gain, there was not a main effect of genotype but there was a significant interaction between genotype and diet (F_1, 33_ = 6.81, *p* = 0.014; Figure 4C) that supports the differences in the growth curves. Splitting the data by diet and analyzing by *t*-test shows that KO females gain significantly more weight than WT females only when on a HFD (T_17_ = 2.63, *p* = 0.018). This indicates that the differences in body weight between KO and WT rats was maintained in rats on a LFD, while a HFD exacerbated the differences in weight. Expectedly, HFD females gained more weight than LFD females (F_1, 31_ = 23.51, *p* = 3.31e-5).

During the first 10 weeks on diet, KO females also ate more than WT (F_1, 13_ = 7.54, *p* = 0.017; Supplementary Figure S1B) and LFD females ate more than HFD (F_1, 13_ = 100.82, *p* = 1.72e-7), with no interaction between genotype and diet. Congruently, over time KO females consumed more calories per week than WT females (F_1,8_ = 6,75, *p* = 0.032; Figure 4D). As with males, HFD females still consumed more kcal than LFD. When we examined the slope of the cumulative energy lines, we found that KO HFD females had a higher energy consumption rate than WT HFD females (F_1, 86_ = 8.17, *p* = 0.0053; Figure 4E) but we did not see a difference in LFD females.

### Male knock-out rats exhibit no difference in fat mass relative to wild-type, despite increased body weight

**FIGURE 5:**
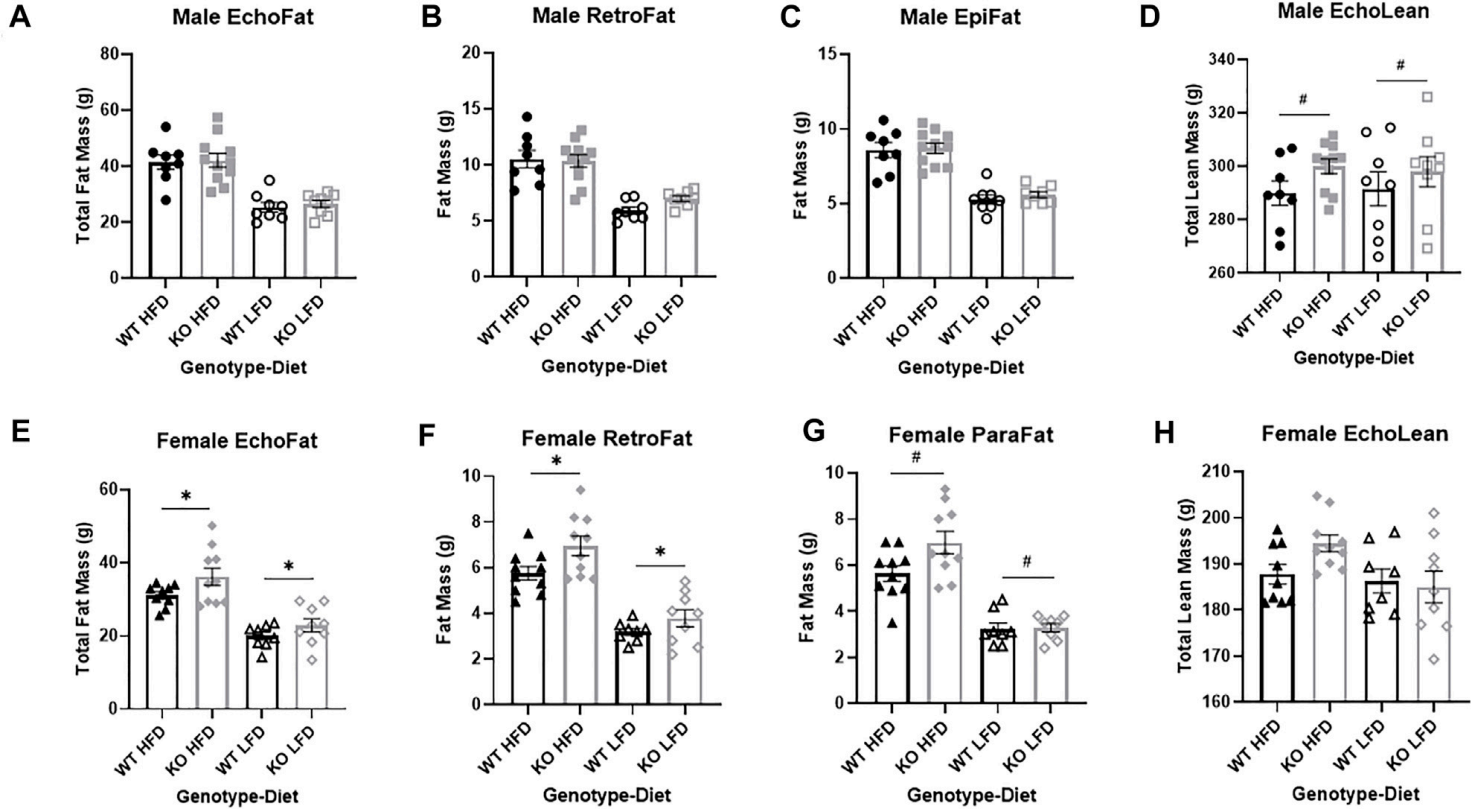
Fat mass and lean mass in wild-type (WT; black) and *Krtcap3* knock-out (KO; grey) rats, for males (top) and females (bottom). After 10 weeks on diet, **(A)** there were no differences in total fat mass using EchoMRI analysis (EchoFat) between WT males (circle) and KO males (square), regardless of diet condition (high fat diet (HFD; filled symbols) and low fat diet (LFD; open symbols)). At euthanasia, there were no differences **(B)** in retroperitoneal fat (RetroFat) mass or in **(C)** Epididymal fat (EpiFat) mass. **(D)** Earlier at EchoMRI analysis there were slight differences in lean mass (EchoLean) between WT males and KO males, but no differences between males on a HFD or a LFD. On the other hand, **(E)** KO females (diamond) on both diets had increased total fat mass using EchoMRI analysis compared to WT females (triangle). At euthanasia, KO females had **(F)** increased RetroFat mass, and **(G)** a trend toward increased parametrial fat (ParaFat) mass. Earlier at EchoMRI analysis, **(H)** there were no differences in lean mass between WT females and KO females in either diet condition. Significance represents main effect of genotype at the following significance levels: ^#^ *p* < 0.1, *p < 0.05, **p < 0.01. Diet effects were also seen as reported in the “Results” section.

After 10 weeks on diet at EchoMRI analysis, there were no differences between WT and KO male total fat mass (Figure 5A), but HFD males had more fat mass than LFD (F_1, 33_ = 54.53, *p* = 1.76e-8). Consistent with EchoMRI analysis, there were no genotype-driven differences in RetroFat (Figure 5B), although HFD males had greater RetroFat than LFD (F_1, 32_ = 52.08, *p* = 3.39e-8). Likewise, there was no difference in gonadal fat between WT and KO males (Figure 5C), but HFD males had increased gonadal fat relative to LFD (F_1, 32_ = 79.99, *p* = 3.23e-10). There were also no differences in OmenFat between WT and KO males, but HFD males had increased OmenFat compared to LFD (F_1, 20_ = 46.688, *p* = 1.22e-6) (data not shown). In the EchoMRI analysis, KO males had a trend toward greater lean mass than WT (F_1, 33_ = 3.14, *p* = 0.086), with no differences in lean mass by diet (Figure 5D). At the end of the study there was no difference in body length by genotype that would explain the increased body weight of KO rats (Supplementary Figure S2A). Brain, heart, and kidney were measured as select lean tissues. There was a trend toward increased brain weight in the KO males relative to WT (F_1, 32_ = 2.95, *p* = 0.096; Supplementary Figure S2B). But there were no significant differences in heart or kidney weight between WT and KO males (Supplementary Figures S2A,B).

### Female knock-out rats have increased fat mass relative to wild-type

After 10 weeks on diet at EchoMRI analysis, KO females had significantly greater total fat mass relative to WT females (F_1, 33_ = 5.32, *p* = 0.028; Figure 5E). As expected, HFD females had more fat mass than LFD (F_1, 33_ = 48.70, *p* = 5.59e-8). At the end of the study, KO females had greater RetroFat relative to WT (F_1, 34_ = 6.01, *p* = 0.020; Figure 5F) and HFD females had greater RetroFat relative to LFD (F_1, 34_ = 71.90, *p* = 6.66e-10). KO females had a trend toward increased gonadal fat relative to WT (F_1, 33_ = 3.38, *p* = 0.075; Figure 5G) and HFD females had significantly higher gonadal fat relative to LFD (F_1, 33_ = 87.46, *p* = 8.43e-11). However, there were no differences in OmenFat by genotype, although HFD females did have increased OmenFat compared to LFD (F_1, 23=_13.214, p = 2e-4) (data not shown). There were no differences in lean mass between WT and KO females at EchoMRI analysis, although HFD females did have greater lean mass than LFD females (F_1, 33_ = 4.79, *p* = 0.036) (Figure 5H). At study conclusion there were no differences in body length between WT and KO females (Supplementary Figure S2E). As with males, select lean tissues were weighed (brain, heart, and kidney). Although there were no genotype-driven differences in brain or kidney weight (Supplementary Figures S2f,H), KO females of both diets had a slightly heavier heart weight than WT females (F_1, 33_ = 4.67, *p* = 0.038; Supplementary Figure S2G).

### Glucose tolerance is similar between wild-type and knock-out rats in both sexes

**FIGURE 6:**
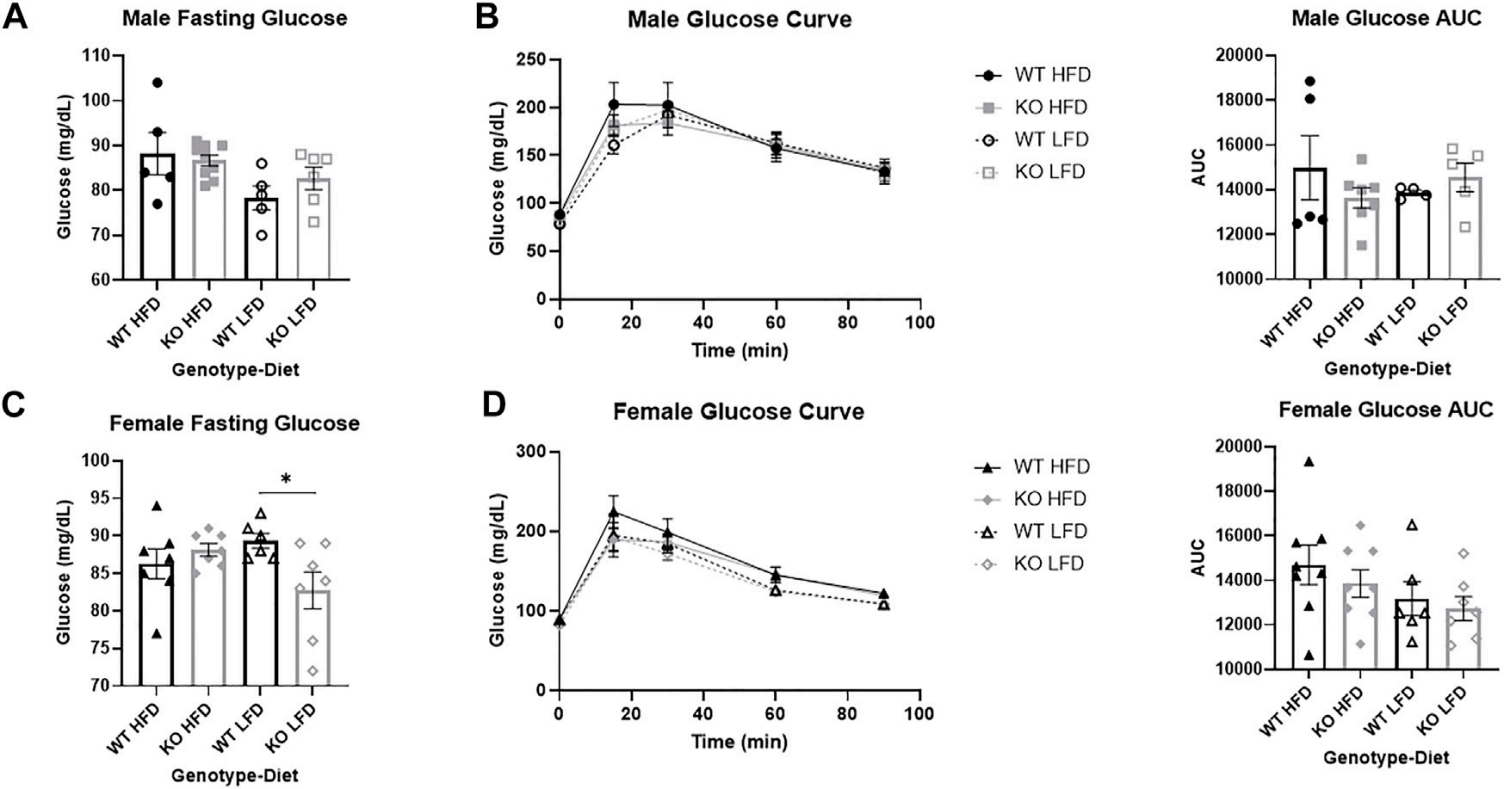
No differences in glucose response between wild-type (WT, black) and *Krtcap3* knock-out (KO, gray) rats. After 12 weeks on diet, male (top) and female (bottom) rats were fasted for 16 h overnight in preparation for an intraperitoneal glucose tolerance test. **(A)** There were no differences in fasting glucose between WT males (circle) and KO males (square), neither on a high-fat diet (HFD; filled) nor a low-fat diet (LFD; empty). **(B)** There were also no significant differences in glucose area-under-the-curve (AUC) between WT and KO males, on either diet condition. **(C)** There was a significant genotype-by-diet interaction for female fasting glucose. While there was no difference in fasting glucose between WT females (triangle) and KO females (diamond) on a HFD, when on a LFD KO females had a lower fasting glucose than WT females. *p < 0.05 for an effect of genotype in the LFD condition. **(D)** There was no significant difference in glucose AUC between WT and KO females. Diet effects were seen as reported in the “Results” section.

There was not a significant effect of genotype on fasting glucose in male rats (Figure 6A), but HFD males had increased fasting glucose relative to LFD (F_1, 22_ = 6.14, *p* = 0.021). There were no differences by genotype or by diet on glucose AUC in males (Figure 6B).

There were no differences in fasting glucose by genotype or by diet in female rats, but there was a genotype by diet interaction (F_1, 24=_5.70, *p* = 0.026) where KO LFD females had lower fasting glucose than WT LFD females (T_11=_2.51, *p* = 0.029; Figure 6C), with no differences in HFD females. While there was no difference in glucose AUC by genotype (Figure 6D), HFD females had a slightly increased glucose AUC relative to LFD (F_1, 26_ = 3.38, *p* = 0.077).

### Male knock-out high-fat diet rats are more insulin resistant than wild-type

**FIGURE 7:**
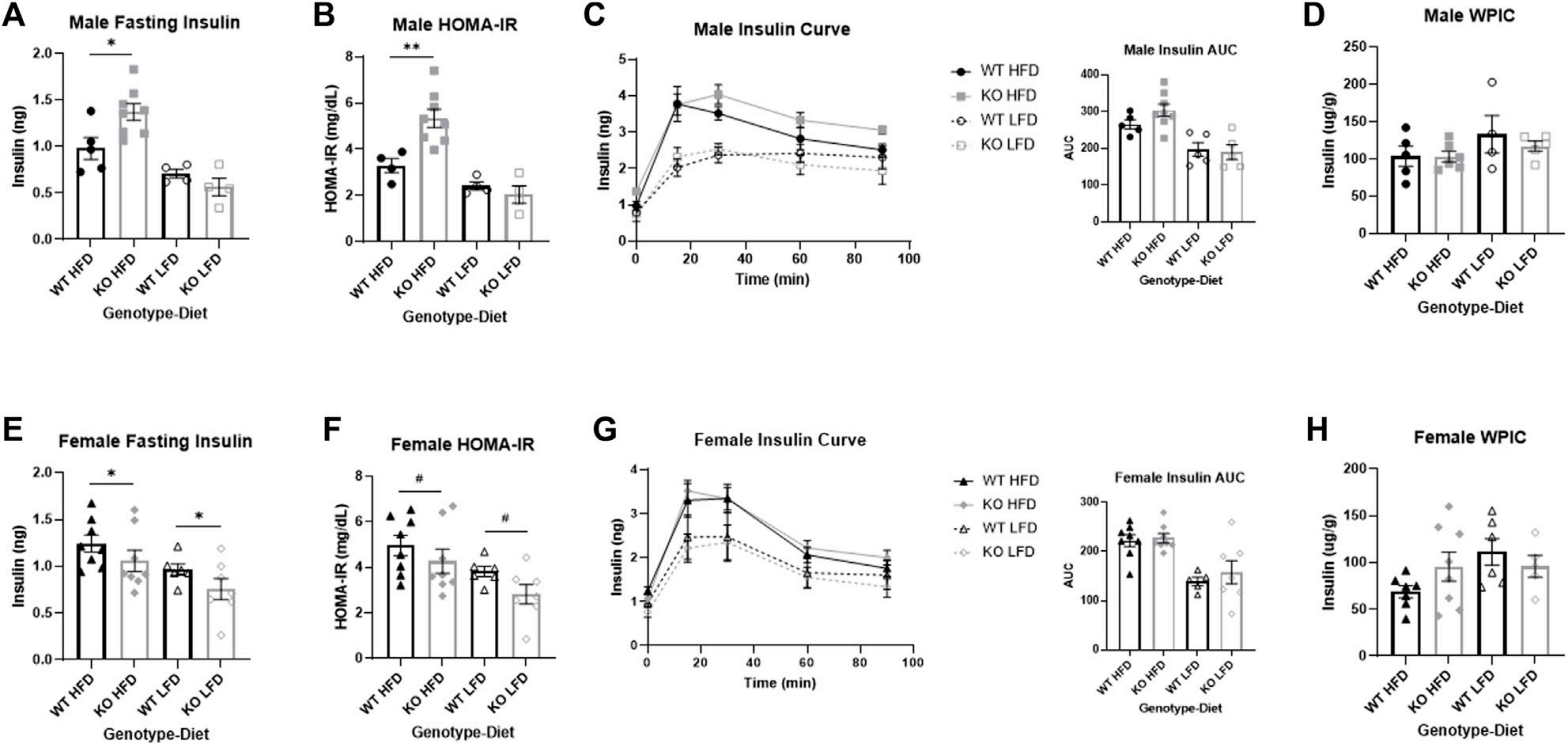
Sex differences in insulin response between wild-type (WT, black) and *Krtcap3* knock-out (KO, gray) male (top) and female (bottom) rats. Insulin was measured in serum collected from the intraperitoneal glucose tolerance test at 12 weeks on diet, and whole pancreas insulin content (WPIC) was measured per gram pancreas collected at euthanasia. **(A)** There was a significant genotype-by-diet interaction in male rats such that KO males (square) had a higher fasting insulin than WT males (circle) only on a high-fat diet (HFD; filled), not a low-fat diet (LFD; empty). *p < 0.05 for an effect of genotype in the HFD condition. **(B)** There was a genotype-by-diet interaction for homeostatic model assessment of insulin resistance (HOMA-IR) in male rats such that KO males had a higher HOMA-IR score than WT males only when on a HFD. **p < 0.01 for an effect of genotype in the HFD condition. **(C)** There were no significant differences in insulin area-under-the-curve (AUC) between WT and KO males. **(D)** There were no significant differences in WPIC between WT and KO males. **(E)** KO females (diamond) on both diets had a lower fasting insulin than WT females (triangle). *p < 0.05 for a main effect of genotype. **(F)** KO females had a trend toward a lower HOMA-IR score compared to WT females. ^#^ *p* < 0.1 for a main effect of genotype. **(G)** There were no differences, however, in insulin AUC between WT and KO females and **(H)** there were no significant differences in WPIC between WT and KO females of either diet. Diet effects are reported in the “Results” section.

There was a significant effect of genotype on fasting insulin in males (F_1, 17_ = 5.97, *p* = 0.026) which was mainly driven by a genotype by diet interaction (F_1, 17_ = 6.82, *p* = 0.018) where KO HFD males had increased fasting insulin relative to WT HFD males (T_11_ = 2.67, *p* = 0.022) with no difference by genotype in LFD males (Figure 7A). As expected, HFD males had greater fasting insulin than LFD (F_1, 17_ = 30.43, *p* = 3.78e-5). There was also a significant effect of genotype on HOMA-IR (F_1, 16_ = 12.23, *p* = 0.003) which was also driven by a significant genotype by diet interaction (F_1, 16_ = 1.16, *p* = 0.0089) where KO HFD males had higher HOMA-IR scores than WT HFD (T_10_ = 3.34, *p* = 0.0074) with no difference by genotype in LFD males (Figure 7B). As expected, HFD males had greater HOMA-IR scores than LFD (F_1, 16_ = 31.59, *p* = 3.83e-5). There was no genotype-driven effect on insulin AUC (Figure 7C), although HFD males had a higher insulin AUC than LFD (F_1, 20_ = 26.63, *p* = 4.76e-5). There was no effect of genotype or diet on WPIC (Figure 7D).

### Female knock-out rats are more insulin sensitive than wild-type

There was a significant effect of genotype on fasting insulin in female rats, but in the opposite direction of the males, with KO females having decreased fasting insulin relative to WT (F_1, 26_ = 4.31, *p* = 0.048; Figure 7E). Similar to males, HFD females had increased fasting insulin relative to LFD (F_1, 26_ = 8.56, *p* = 0.0071). There was a nearly significant main effect of genotype on HOMA-IR (F_1, 26_ = 4.12, *p* = 0.053; Figure 7F) with increased HOMA-IR in WT females relative to KO, and as expected increased HOMA-IR in HFD females relative to LFD (F_1, 26_ = 8.83, *p* = 0.0063). As with males, while there was no difference in insulin AUC by genotype (Figure 7G), HFD females did have a greater insulin AUC than LFD (F_1, 26_ = 9.04, *p* = 0.0058). There was no effect of genotype or of diet for WPIC (Figure 7H).

### No differences in serum or liver lipids between wild-type and knock-out males

**FIGURE 8:**
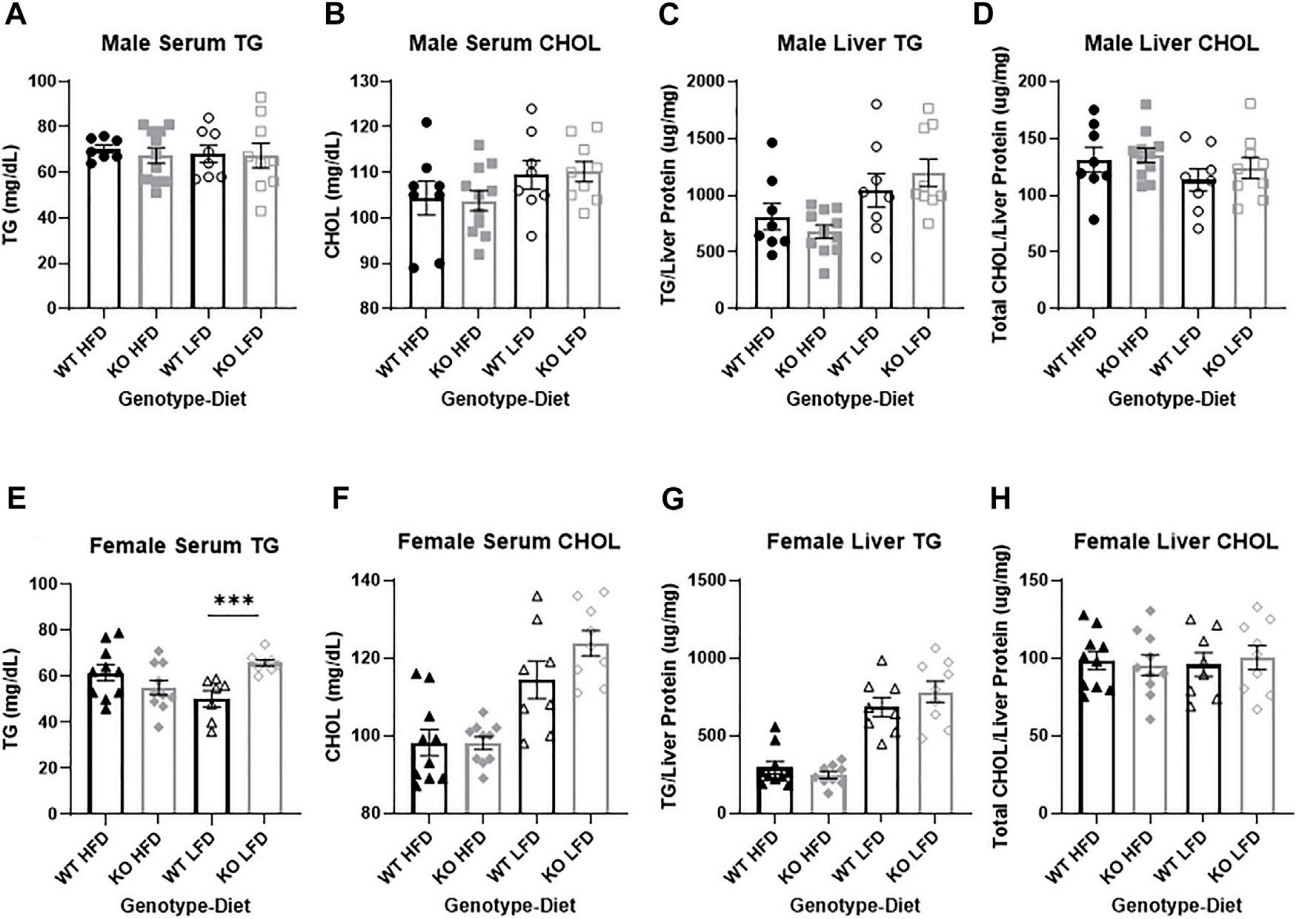
No differences in liver metabolic health between wild-type (WT; black) and *Krtcap3* knock-out (KO; gray). Triglycerides (TG) and total cholesterol (CHOL) were measured in serum collected from male (top) and female (bottom) rats. There was no difference between WT males (circle) and KO males (square) on a high-fat diet (HFD; filled) or a low-fat diet (LFD; empty) for **(A)** serum TG or **(B)** serum CHOL. TG and CHOL were also measured in liver tissue of male rats, with no differences between WT and KO males for **(C)** liver TG or **(D)** liver CHOL. **(E)** There was a genotype-by-diet interaction in serum TG in female rats, such that while there was no difference between WT (triangle) and KO females (diamond) on a HFD, KO females had increased serum TG compared to WT on a LFD. ***p < 0.001 for an effect of genotype in the LFD condition. **(F)** There were no differences in serum CHOL between WT and KO females. There were no differences in **(G)** liver TG nor **(H)** liver CHOL between WT and KO females. Diet effects are reported in the “Results” section.

There were no significant differences by genotype or by diet in serum triglycerides in male rats (Figure 8A). While there was no effect of genotype on serum total cholesterol (Figure 8B), HFD males had lower cholesterol than LFD (F_1, 33_ = 4.46, *p* = 0.042). Similarly, there were no differences in liver triglycerides between WT and KO males (Figure 8C), but HFD males had lower liver triglycerides than LFD (F_1, 33_ = 12.46, *p* = 0.0013). There were no differences by genotype or by diet in liver cholesterol (Figure 8D). The lack of differences in liver lipids by genotype were true whether lipids were normalized by liver weight or liver protein. Liver metabolites in the serum (ALP, AST, ALT, and albumin) were also analyzed, with no significant effects driven by genotype (see Supplementary Figures S2A–E).

### No pathological differences in serum or liver lipids between wild-type and knock-out females

There were no differences by genotype or by diet in serum triglycerides for female rats but there was a genotype by diet interaction (F_1, 31=_12.01, *p* = 0.0016) where only KO LFD females had higher triglycerides relative to WT LFD females (T_13=_14.35, *p* = 0.00079; Figure 8E), with no difference in HFD females. Genotype did not affect serum total cholesterol (Figure 8F), but HFD females had lower cholesterol than LFD (F_1, 34_ = 39.75, *p* = 3.47e-7). Similarly, there were no differences in liver triglycerides by genotype (Figure 8G), but HFD females had lower liver triglycerides relative to LFD (F_1, 33_ = 96.77, *p* = 2.44e-11). As with the males, there were no differences by genotype or diet in liver cholesterol (Figure 8H). The lack of difference in liver lipids by genotype were true whether lipids were normalized by liver weight or liver protein. Liver metabolites in the serum (ALP, AST, ALT, and albumin) were also analyzed, with no significant effects driven by genotype (see Supplementary Figures S2F–J).

### No differences in adipocyte size or number between wild-type and knock-out high-fat diet females

**FIGURE 9:**
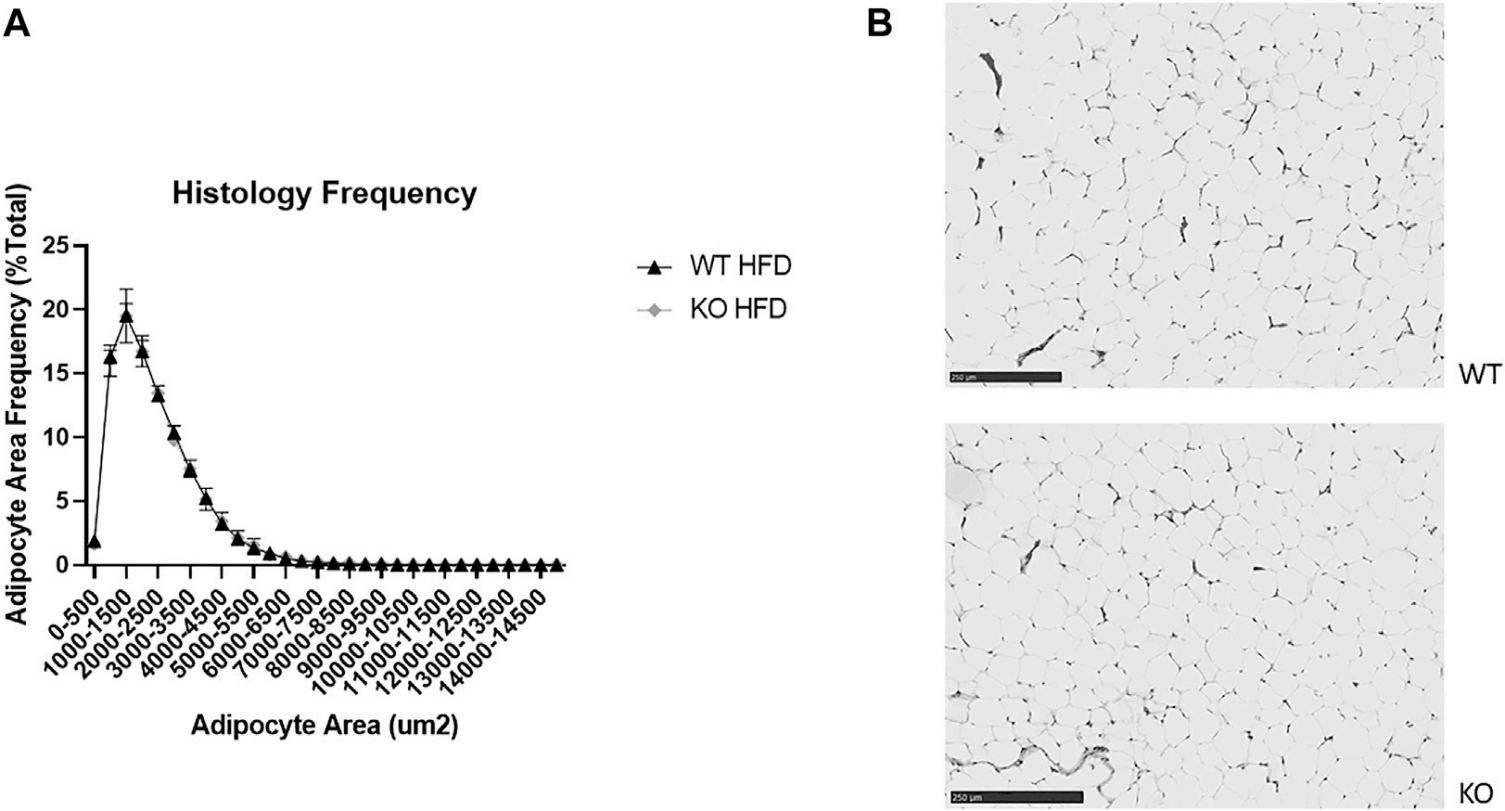
No difference in adipocyte size or number between wild-type (WT; black filled triangle) and *Krtcap3* knock-out (KO; gray filled diamond) female rats. Sections of retroperitoneal fat (RetroFat) were taken from representative WT and KO females on a high-fat diet (HFD) and stained for hematoxylin and eosin. **(A)** There were no differences in the frequency distributions of adipocyte area between WT and KO females, as shown by **(B)** representative adipocytes between WT (top) and KO (bottom) rats. Images were re-colored to grayscale and sharpened by 25%.

As expected, there were few very large adipocytes in both WT and KO rats, with a significant effect of adipocyte bin size (F_29, 269_ = 321, p = 2e-16), but surprisingly there were no differences by genotype in the adipocyte area frequency distributions (Figure 9A). Representative images demonstrate this lack of difference (Figure 9B).

### Adult wild-type females have very little liver Krtcap3 expression compared to wild-type males

Although there were no apparent sex differences in *Krtcap3* expression in young rats, at the end of the study, we found that adult WT females have approximately 15% of the adult WT male *Krtcap3* expression in the liver (F_1, 10_ = 160.26, *p* = 1.76e-7, data not shown). There was not a significant effect of diet on *Krtcap3* expression in adult WT rats.

## Discussion

We demonstrate that *Krtcap3* plays a role in body weight in both sexes, but that only females exhibit increased eating and adiposity. Despite increased food intake, body weight, and fat mass, KO females had increased insulin sensitivity and no differences in adipocyte size, suggesting a metabolically healthy phenotype. In contrast, while we saw increased body weight in KO male rats, we were unable to detect significant differences in eating behavior or fat mass compared to WT males. However, KO males had increased insulin resistance compared to WT males when consuming a HFD. This is the first study to demonstrate a physiological role of *Krtcap3* expression in adiposity, showing its role in food intake and insulin sensitivity and establishing important sex and environmental differences.

*Krtcap3* has previously been identified as a candidate adiposity gene in both humans and rats in GWAS analyses (Chen et al., 2018; Keele et al., 2018). Little else is known about the underlying function of *Krtcap3*, with previous work showing a role in sheep wool production and some human cancers (Wang et al., 2014; Giuliani et al., 2016; Fujiwara et al., 2018; Wang et al., 2020). The protein KRTCAP3 is predicted to be an integral membrane protein (www.genecards.org), but no validation or functional studies for its role in adiposity have been conducted. Publically available data show that in humans, *Krtcap3* is highly expressed in the digestive tract, the male and female sex organs, the pituitary and thyroid glands, the lungs, the pancreas, and the skin (https://gtexportal.org). Beyond this knowledge, *Krtcap3* has been poorly studied, in obesity and overall.

In the current work, we found that at 6 weeks of age, KO females on the WKY background were heavier than WT controls, and these differences became more pronounced when the rats aged when on a HFD. That said, KO females on both diets had increased fat mass relative to WT. We propose that increased food intake and energy consumption drives the body weight and adiposity phenotype in the KO females. In support of this discovery, findings from human GWAS for BMI are enriched in the central nervous system and may play a role in satiety signaling (Locke et al., 2015; Loos, 2018; Turcot et al., 2018; Yengo et al., 2018). *Krtcap3,* however, has low expression in both the hypothalamus and hippocampus (https://gtexportal.org), the brain regions associated with food intake (Ahima and Antwi, 2008). But *Krtcap3* is highly expressed in the gut, so one possibility is that *Krtcap3* may play a role in the gut-brain axis to affect food intake (Sam et al., 2012). Future work is necessary to investigate this hypothesis.

Although the *Krtcap3*-KO females exhibited increased adiposity, these rats did not show worsened metabolic health. Specifically, there were not any pathological genotype-driven differences in glucose or lipid metabolism, but KO female rats were more insulin sensitive compared to WT counterparts. Furthermore, while adipocyte hypertrophy is associated with human obesity (Verboven et al., 2018), there were no differences in adipocyte area between WT and KO females. Given that KO females have greater fat mass than WT females but no difference in adipocyte size, this would suggest that KO females have increased adipogenesis, leading to adipocyte hyperplasia. Adipocyte hyperplasia over adipocyte hypertrophy has generally been associated with a metabolically healthy phenotype (Longo et al., 2019; Vishvanath and Gupta, 2019), which supports the other metabolic health data from this study. These data point to *Krtcap3* being a gene that increases body weight and fat mass in female rats without the associated co-morbidities. Interestingly, recent human GWAS have identified genetic variants that increase body fat percentage but are protective against metabolic complications (Loos and Kilpelainen, 2018). We posit that lack of *Krtcap3* may promote weight gain without diminishing metabolic health, at least in females.

In male rats, we found that KO rats were heavier than WT controls at 6 weeks of age, and that this body weight difference was maintained throughout the study. This is supported by differences in weight by genotype at euthanasia, but no differences in weight gain between WT and KO males. Unexpectedly, we did not see increased adiposity in the male KO compared to WT rats, nor were there significant eating differences. The body weight difference is most likely explained by the slight increase in lean mass in KO males compared to WT males, though neither body length nor the select tissues we collected at euthanasia explain the difference. The lack of an adiposity difference was surprising, as *Krtcap3* was originally identified as a candidate gene in male rats (Keele et al., 2018). Despite not seeing an adiposity phenotype, we found that KO HFD males had hyperinsulinemia and insulin resistance compared to WT controls, with no differences in LFD males. Similar to females, there were no genotype-driven differences in lipid metabolism.

As the original study identified *Krtcap3* expression in liver (Keele et al., 2018), we anticipated that KO rats would have increased serum and liver lipids. Surprisingly, there were no pathological genotype-driven differences in these measures in either male or female rats. These data suggest that while liver *Krtcap3* expression is associated with increased fat mass, its role in the liver is likely not driving the increased adiposity. This is supported by the finding that, despite relatively high *Krtcap3* expression in the liver of adult male rats, there was much lower expression in the livers of adult females, in whom we saw an adiposity phenotype. In humans, *Krtcap3* is highly expressed in tissues besides the liver, presenting different tissues of action to consider. As stated above, *Krtcap3* may play a role in the gut-brain axis to impact satiety. It is also possible that *Krtcap3* may be acting in the sex organs, which may help explain some of the sex differences seen here.

Sex differences in obesity have been well-established in rodents and humans (Palmer and Clegg, 2015; Link and Reue, 2017; Schorr et al., 2018; Lumish et al., 2020) and may lend support to the striking sex differences seen here. The differences we see in phenotype may be explained by *Krtcap3* having a larger effect size in females relative to males, which is supported by literature on sex-specific obesity loci, where loci explain more variance in women than men (Heid et al., 2010; Shungin et al., 2015). Future replication studies to increase the *n* may allow us to see an effect in males. We did see a slight elevation in cumulative energy consumption in KO HFD males compared to WT HFD males toward the end of the study, which may also indicate a need to lengthen the amount of time the rats are on diet to see an effect in males. The second explanation is that *Krtcap3* may interact with gonadal hormones to cause sex differences (Link and Reue, 2017). Sex steroid hormones are known to play a role in food intake, fat deposition, and even diabetes susceptibility (Palmer and Clegg, 2015; Link and Reue, 2017; Tramunt et al., 2020). Estrogens in particular can suppress appetite, alter fat deposition, and protect against insulin resistance (Palmer and Clegg, 2015; Tramunt et al., 2020). An interaction between *Krtcap3* and sex hormones may explain the different adiposity and insulin phenotypes between male and female rats in the present study. Future studies are necessary to explore these hypotheses.

At a glance, the differences in adiposity and metabolic health described here may seem small, but it is unsurprising that knocking-out *Krtcap3* had a modest effect on adiposity and metabolism. *Krtcap3* was originally identified as part of a QTL for RetroFat on rat chromosome 6 with an effect size of 11.05% (Keele et al., 2018). Fine-mapping of this QTL supported multiple independent signals, of which *Krtcap3* was only one (Keele et al., 2018). This corresponds to what we would expect in human obesity. The majority of patients suffer from polygenic obesity: multiple common variants individually have small effects but can overall increase a patient’s susceptibility to weight gain in an obesogenic environment, particularly in patients with an already high BMI (Abadi et al., 2017; Bouchard, 2021). And as reviewed elsewhere, though common SNPs as a whole may account for two-thirds of BMI heritability, each SNP will individually only affect weight to a small degree (Bouchard, 2021). While there appears to only be a modest effect on body weight, fat mass, and insulin sensitivity in these rats, these data demonstrate that expression of *Krtcap3* has an effect on adiposity phenotypes. Specifically, in female rats on a HFD, decreasing expression of *Krtcap3* led to a weight gain of a little over 15 g (approximately a 7% increase in final body weight). Plus, KO females had greater total and visceral fat mass even without the obesogenic conditions of a HFD. In male rats, there was a significant increase in insulin resistance when decreased *Krtcap3* expression was combined with a HFD, despite there being no difference in adiposity. These data confirm that *Krtcap3* expression impacts adiposity in rats and should be considered for future studies.

Because so little is known about the KRTCAP3 protein, including where it is acting to impact adiposity, the current studies do not lend themselves to understanding the underlying mechanism. By being the first to confirm *Krtcap3* as a novel obesity gene, however, this work sets the stage for future mechanistic studies. Future work will assess differences in *Krtcap3* expression in multiple tissues between the sexes to help determine the tissue of action and help explain the sex-dependent adiposity outcomes described here. Once a tissue of action is identified, future work will allow us to identify potential protein partners and determine pathways of action.

This work validates *Krtcap3* as a novel gene for adiposity. We show that *Krtcap3-*KO female rats exhibit increased body weight, food intake, and fat mass with improved metabolic health. In contrast, *Krtcap3-*KO male rats exhibit increased body weight with worsened metabolic health on a HFD, but no change in fat mass or food intake. This work sets the stage for future functional and mechanistic studies. Further work will explore the sex differences in expression and phenotype, identify where *Krtcap3* is acting to influence food intake, and establish a mechanism to understand its impact on adiposity.

## Data Availability

The data presented in the study are deposited in the FigShare repository, accession number 10.6084/m9.figshare.19750672.
