# Supplementary figures and images for: Keratinocyte-associated protein 3 plays a role in body weight and adiposity with differential effects in males and females
Source: Front Genet. 2022 Sep 21;13:942574. doi: 10.3389/fgene.2022.942574 (PMC9535360; doi:10.3389/fgene.2022.942574)

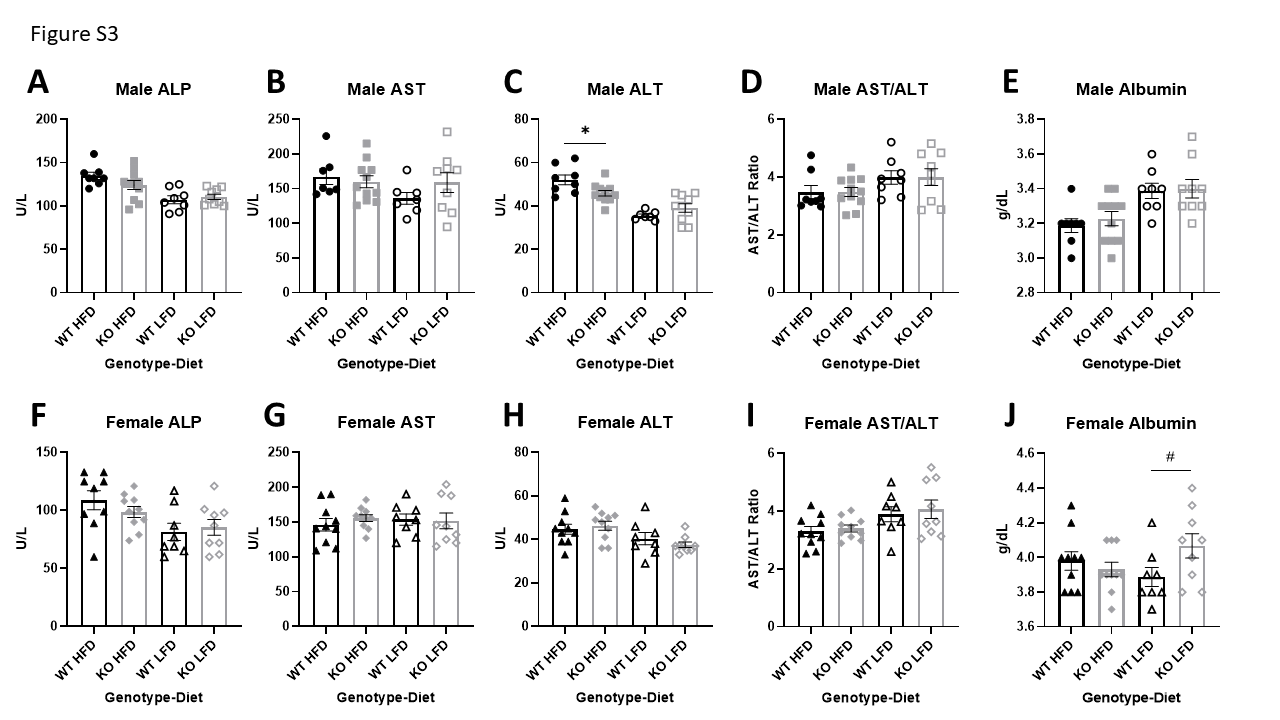

Supplement: Supplementary file 1 [file Image3.TIF]

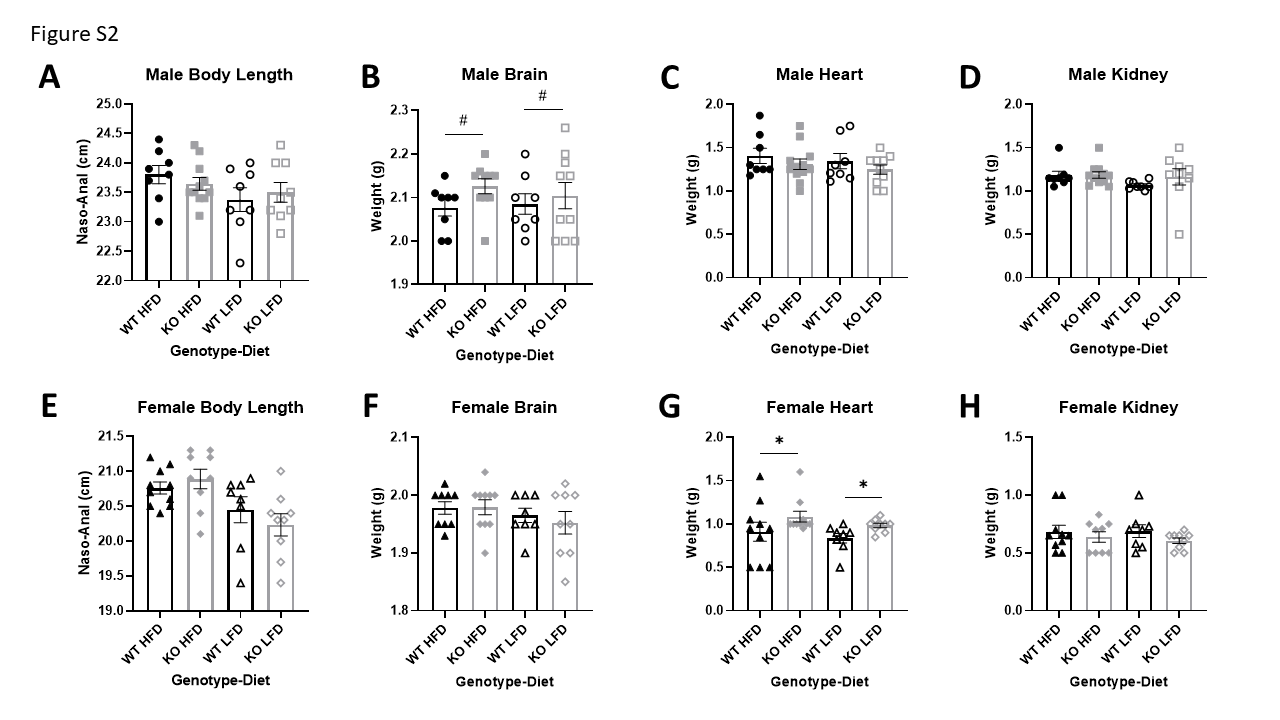

Supplement: Supplementary file 2 [file Image2.TIF]

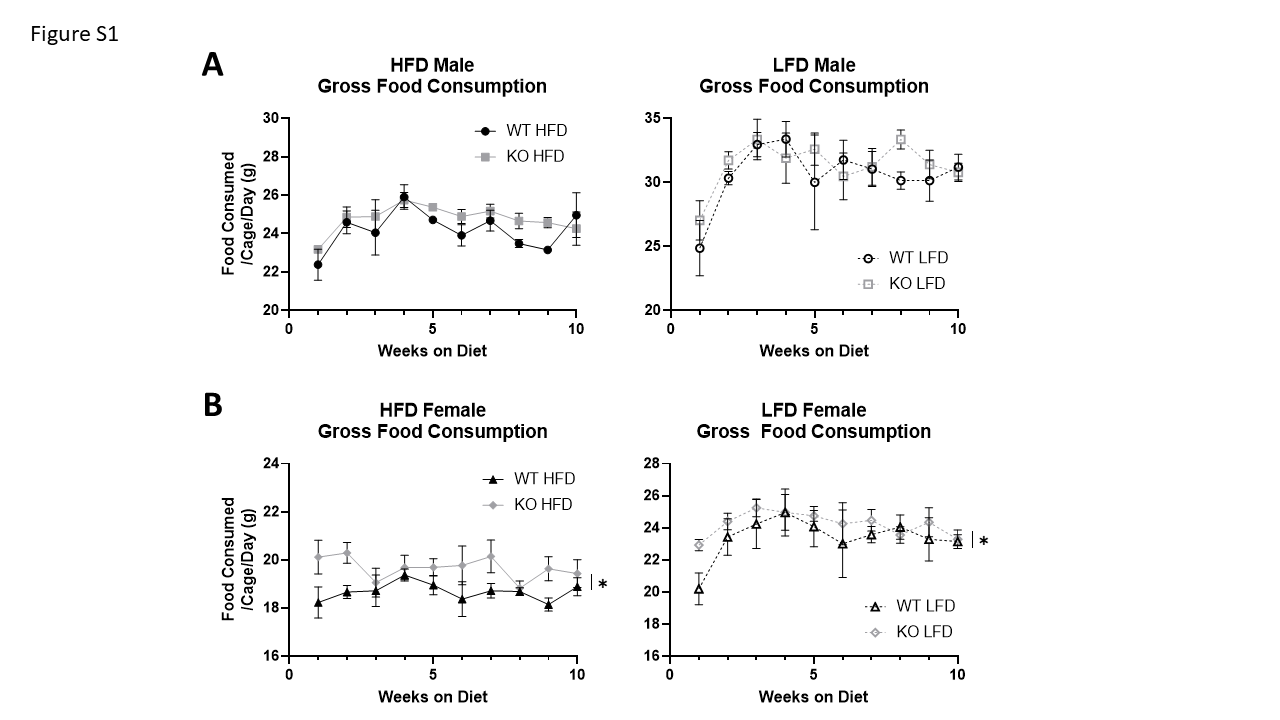

Supplement: Supplementary file 3 [file Image1.TIF]
