# Supplementary material for: Keratinocyte-associated protein 3 plays a role in body weight and adiposity with differential effects in males and females
Source: Front Genet. 2022 Sep 21;13:942574. doi: 10.3389/fgene.2022.942574 (PMC9535360; doi:10.3389/fgene.2022.942574)
Supplement: Supplementary file 4 [file DataSheet1.PDF]

Supplementary Material

1 Supplementary Results

Females, but not males, show genotype-driven differences in food intake

There were no differences in gross food consumption between WT and KO male rats (**Figure S1A**), but LFD males ate significantly more than HFD males ($F_{1, 11}$=120.18, p=2.93e-7). In contrast, KO females ate more than WT ($F_{1, 13}$=7.54, p=0.017; **Figure S1b**) and LFD females ate more than HFD ($F_{1, 13}$=100.82, p=1.72e-7), with no interaction between genotype and diet.

No pathological genotype-driven differences in select lean organ weights between WT and KO males

There were no differences in body length between WT and KO rats (**Figure S2a**), but HFD males were slightly longer than LFD males ($F_{1, 32}$=3.34, p=0.077). KO male rats of either diet had a slightly elevated brain weight compared to WT males ($F_{1, 32}$=2.95, p=0.096; **Figure S2b**), but there were no differences by diet. There were no differences in heart weight by genotype or by diet (**Figure S2c**). There were no differences in kidney weight either by genotype or by diet (**Figure S2d**).

No pathological genotype-driven differences in select lean organ weights between WT and KO females

While there no differences in body length between WT and KO rats (**Figure S2e**), HFD females were significantly longer than LFD females ($F_{1, 32}$=11.4, p=0.002). There were no differences in brain weight of female rats either by genotype or diet (**Figure S2f**). There was, however, a genotype effect in heart weight, where KO females of either diet had a slightly heavier heart than WT females ($F_{1, 33}$=4.67, p=0.038; **Figure S2g**), but there was no difference by diet. As with males, there were no differences in kidney weight by genotype (**Figure S2h)**.

No pathological genotype-driven differences in liver metabolites between WT and KO males

There were no differences by genotype in ALP or AST of male rats (**Figure S3a-b**). There was a diet effect where HFD rats had higher ALP than LFD rats ($F_{1,31}$=23.42, p=3.4e-5), but there were no differences by diet in AST. There was an interaction between genotype and diet for ALT ($F_{1, 30}$=8.05, p=0.008; **Figure S3c**), and follow-up analysis showed that KO HFD males had lower ALT than WT (T_{17}=-2.53, p=0.022), with no differences in LFD males. HFD males also had increased ALT compared to LFD males ($F_{1, 30}$=39.07, p=6.93e-7). Driven by the effect in ALT, there was a diet effect in the AST/ALT ratio ($F_{1, 31}$=5.8, p=0.022; **Figure S3d**), but no effect of genotype. Finally, HFD males had decreased albumin compared to LFD males ($F_{1, 31}$=14.66, p=5.9e-4; **Figure S3e**), but no differences between WT and KO males.

No pathological genotype-driven differences in liver metabolites between WT and KO females

HFD females had increased ALP compared to LFD females ($F_{1, 32}$=8.92, p=0.005; **Figure S3f**), with no differences between WT and KO females. There were no effects of genotype or diet in AST (**Figure S3g**), but there was a diet effect in ALT, where HFD females had higher ALT than LFD females ($F_{1, 32}$=10.39, p=0.003; **Figure S3h**). Correspondingly, HFD females had a lower AST/ALT ratio than LFD females ($F_{1, 33}$=8.11, p=0.008; **Figure S3i**), with no differences by genotype. There was an interaction between genotype and diet for albumin, but follow up analyses showed the KO LFD females had only a slightly higher albumin content than WT (T_{15}=1.96, p=0.068; **Figure S3j**) with no significant differences in HFD females.

2 Supplementary Tables

Krtcap3 Genotyping	ACCCTCTTGCGACGCTCACCAGTA	GCGTCTGCGCCTTCGGTAAGTT
β-actin (qPCR)	TGAGGTAGTCTGTCAGGTCCCG	ACCACTGGCATTGTGATGGACT
Krtcap3 (qPCR)	GTTACTGTTGTGTGGCTGCA	AGCACCTCCTGTCCTAAACC
GAPDH (qPCR)	CATGGAGAAGGCTGGGGCTC	AACGGATACATTGGGGGTAG

Supplementary Table 1. Primer sequences. 5’ → 3’

	LFD (D12450J)	HFD (D12492)
Protein	20	20
Carbohydrate	70	20
Fat (Lard + Soybean Oil)	10	60
Total kcal/g	3.82	5.21

Supplementary Table 2. Dietary composition in %kcal. LFD, low-fat diet; HFD, high-fat diet.

<table>
<tr><th>Sex-Genotype-Diet</th><th>Wean</th><th>6-week</th><th>EchoMRI</th><th>IPGTT</th><th>Sac</th><th>WPIC</th></tr>
<tr><td>M WT HFD</td><td rowspan="2">14</td><td rowspan="2">16</td><td>8</td><td>5</td><td>8</td><td>4</td></tr>
<tr><td>M WT LFD</td><td>8</td><td>5</td><td>8</td><td>4</td></tr>
<tr><td>M KO HFD</td><td rowspan="2">18</td><td rowspan="2">22</td><td>11</td><td>9</td><td>11</td><td>6</td></tr>
<tr><td>M KO LFD</td><td>9</td><td>6</td><td>9</td><td>5</td></tr>
<tr><td>F WT HFD</td><td rowspan="2">16</td><td rowspan="2">18</td><td>10</td><td>8</td><td>10</td><td>8</td></tr>
<tr><td>F WT LFD</td><td>8</td><td>6</td><td>8</td><td>6</td></tr>
<tr><td>F KO HFD</td><td rowspan="2">17</td><td rowspan="2">19</td><td>10</td><td>8</td><td>10</td><td>8</td></tr>
<tr><td>F KO LFD</td><td>9</td><td>7</td><td>9</td><td>5</td></tr>
</table>

Supplementary Table 3. Number of rats per experimental procedure. Male (M), Female (F), wild-type (WT), knock-out (KO), high-fat diet (HFD), low-fat diet (LFD). Experimental procedures include wean weight (Wean), six-week body weight (6-week), EchoMRI analysis (EchoMRI), intraperitoneal glucose tolerance test (IPGTT), euthanasia (Sac), and whole pancreas insulin content (WPIC).

Sex	Phenotype	Type of Transformation	Normality/Variance
Male	Fasting Glucose	Reciprocal root	Normality & Variance
	Glucose AUC	Natural log	Normality
	Liver TG	Natural log	Normality
Female	ParaFat	Natural log	Normality
	Fasting Glucose	^4	Normality
	Serum CHOL	Natural log	Normality
	Liver TG	Natural log	Normality

Supplementary Table 4. Data transformations. TG, triglycerides; ParaFat, parametrial fat; CHOL, cholesterol. Phenotypes not listed had a normal distribution and variance and did not need to be transformed.

3 Supplementary Figures

Figure S1

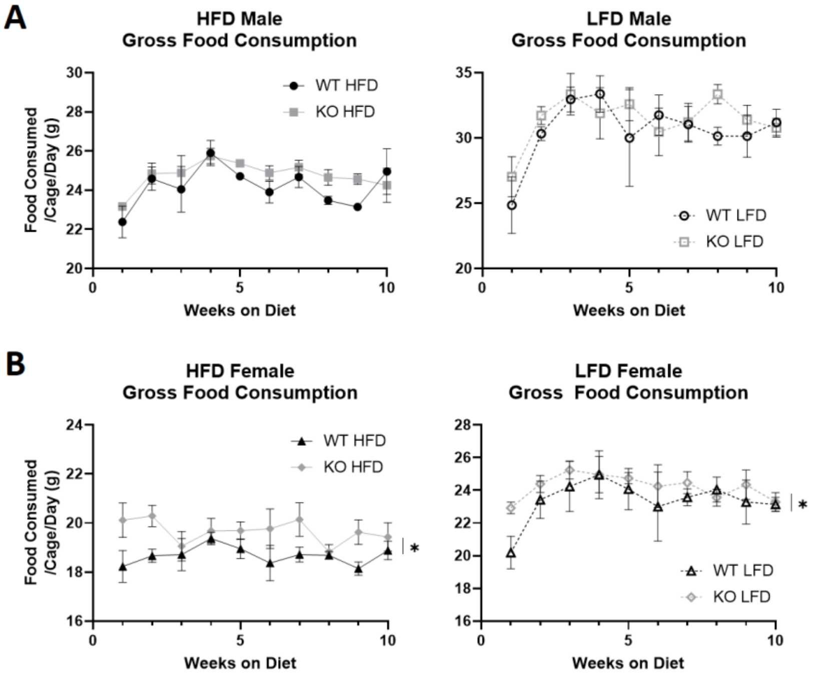

Supplementary Figure 1. Gross food consumption between wild-type (WT; black) and *Krtcap3* knock-out (KO; gray) male and female rats. Male data is displayed in the top row, while female data in the bottom row. To highlight genotype-driven differences, figures are separated by diet, high-fat diet (HFD; filled) and low-fat diet (LFD; empty). (A) There were no differences in eating behavior between WT (circle) and KO males (square) on either diet. (B) There was a main effect of genotype over time on cage food intake for females, where KO females (diamond) consumed more food than WT females (triangle). There were no interactions with diet. *$p < 0.05$ represents a main effect of genotype over time across both diets.

Figure S2

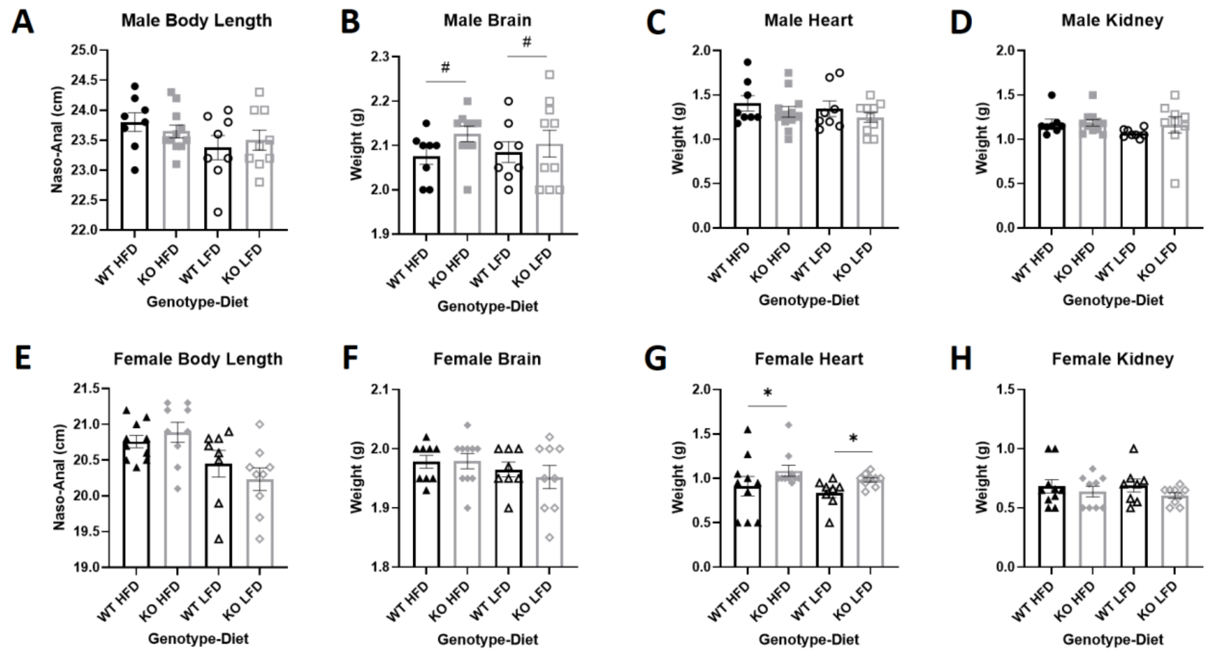

Supplementary Figure 2. Body length and lean tissue mass between wild-type (WT; black) and *Krtcap3* knock-out (KO; gray) male and female rats. Male data is displayed in the top row, while female data in the bottom row. (A) There was no difference in body length between WT males (circle) and KO males (square) whether on a high-fat diet (HFD; filled) or low-fat diet (LFD; empty). (B) KO males on either diet had a slightly heavier brain than WT males, where #$p < 0.01$ represents a main effect of genotype. There were no differences by genotype in either (C) heart weight or (D) kidney weight. (E) There was no difference in body length between WT females (triangle) and KO females (diamond) nor (F) in brain weight. (G) KO females had a significantly heavier heart weight than WT females, where *$p < 0.05$ represents a main effect of genotype. (H) There were no differences in kidney weight between WT and KO females. Diet differences are in the "Supplementary Results" section.

Figure S3

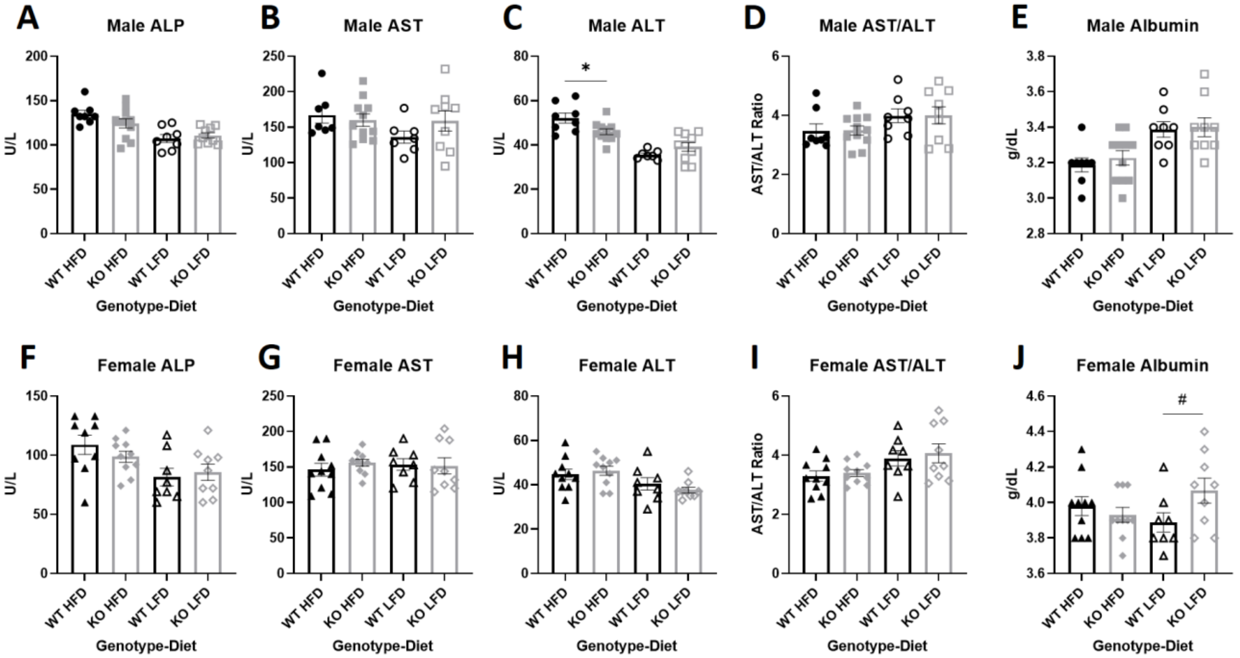

Supplementary Figure 3. Serum metabolic markers of liver health show no pathological differences between wild-type (WT; black) and *Krtcap3* knock-out (KO; gray) rats. Male data is displayed in the top row, while female data in the bottom row. Markers of liver health include alkaline phosphatase (ALP), aspartate transaminase (AST), alanine aminotransferase (ALT), the ratio of AST to ALT (AST/ALT), and albumin. There were no differences in (**A**) ALP between WT (circle) and KO males (square) on either a HFD (filled-in) nor LFD (empty). (**B**) There were also no differences in AST by genotype, though (**C**) WT HFD males had slightly elevated ALT compared to KO HFD males, with no effect in LFD males. *$p < 0.05$ represents an effect in only the HFD condition. Despite this difference, (**D**) there are no significant differences by genotype in the ratio between AST/ALT, and (**E**) there are no significant differences by genotype for albumin. There are no differences between WT (triangle) and KO females (diamond) for (**F**) ALP, (G) AST, (H) ALT, nor the (I) AST/ALT ratio. (J) There was an interaction between genotype and diet for female albumin, but further analyses show only a trend of increased albumin in KO LFD females compared to WT. #$p < 0.1$ represents and effect in only the LFD condition. Diet differences are in the "Supplementary Results" section.
